# Assessing the health status of migrants upon arrival in Europe: a systematic review of the adverse impact of migration journeys

**DOI:** 10.1186/s12992-024-01075-3

**Published:** 2024-09-27

**Authors:** Cristina Canova, Lucia Dansero, Cinzia Destefanis, Clara Benna, Isabella Rosato

**Affiliations:** 1 Unit of Biostatistics, Epidemiology and Public Health (UBEP), Department of Cardiac, Thoracic, Vascular Sciences and Public Health, University of Padua, Padua, Italy; 2 Centre for Biostatistics, Epidemiology and Public Health (C-BEPH), Department of Clinical and Biological Sciences, University of Turin, Orbassano, Italy; 3 Department of Surgery Oncology and Gastroenterology, University of Padua, Padua, Italy

**Keywords:** Migration journey, Systematic review, Europe, Traumatic events, Mental health

## Abstract

**Background:**

Numerous studies have explored the impact of pre- and post-migration factors on the overall health of migrant populations. The objective of this study is to enhance our understanding of additional determinants affecting migrants' health by examining the impact of the migration phase and related journeys in the European context.

**Methods:**

We conducted a systematic review of studies published in the MEDLINE, Embase, and Scopus databases from 2003 up to January 5, 2024. We included observational studies reporting information on the health status of migrant populations recorded upon arrival in a country situated in Europe, and on the transit phase, including specific risk factors experienced during the journey or its characteristics. Title and abstract screening were performed using active learning techniques provided by ASReview software. The results of the included studies were presented qualitatively, with a focus on publications that formally assessed the association between the journey and the investigated health outcomes. The systematic review was registered on PROSPERO, CRD42024513421.

**Results:**

Out of 11,370 records screened, we ultimately included 25 studies, all conducted since 2017. Most adopted a cross-sectional design and a quantitative approach, with relatively small sample sizes. The majority of the studies were conducted in Serbia and Italy. Only 14 of them formally assessed the association between different exposures in the transit phase and health outcomes, including mental health, well-being and quality of life, infectious and non-communicable diseases.

**Conclusion:**

Epidemiological research focusing on the transit phase in Europe remains limited, with few available studies facing challenges related to data collection, study design and analysis, thereby limiting the interpretability and generalisability of their results. These findings underscore the need for action, prompting the development of adequate and feasible strategies to conduct additional studies focusing on migrant populations during migration journeys.

**Supplementary Information:**

The online version contains supplementary material available at 10.1186/s12992-024-01075-3.

## Introduction

Driven by various geopolitical, economic and social factors, the dimensions of migration flows in European countries have increased in recent years [1]. A notable number of migrants, escaping from conflict-ridden nations such as Syria, Afghanistan, and Somalia, have marked the onset of what is commonly referred to as the “European migration crisis” [2, 3]. In 2015, more than 65 million people migrated towards Europe, posing an unprecedented challenge for European countries [4]. According to the World Health Organization (WHO), European regions host approximately 36% of the global international migrant population [5]. In 2023, almost 293,000 migrants arrived in Europe [6] through one of the main migratory routes: the Western Mediterranean, Central Mediterranean, and Eastern Mediterranean/Balkan routes [7–9].

These diverse populations are commonly referred to as "migrants", a term defined by the International Organization for Migration (IOM) to describe all individuals who move across an international border or within a state away from their habitual place of residence, regardless of legal status, whether the movement is voluntary or involuntary, the causes of the movement, or the length of the stay [10].

The relationship between migration and health is extremely intricate. On the one hand, migrating from a context of conflict and extreme poverty could potentially improve health and social standing; on the other hand, the migration journey itself could constitute a significant health risk [11]. Migratory status can introduce complexity, leaving migrants and refugees more vulnerable to specific health factors depending on the various stages of their displacement. During migration journeys, migrants can suffer from hunger and thirst, experience extortion and robbery, endure maltreatment, violence, and torture [12]. In some cases, they also report experiences of arrest and deportation. Several psychological and physical issues can be related to the dangerous experiences of the migration transit phase [13, 14].

Various studies have explored the health status and well-being of migrant populations both before and after the completion of their journeys, in their countries of origin and destination in the post-migration phase [15–17]. However, there is a gap in the understanding of migrants’ health during the migration journey [18], which may be attributed to challenges in data collection, the lack of organized medical records, and the fragmentation of information collected across different countries during the transit process [19].

To date, there appears to be no systematic review specifically conducted addressing the health status and associated risks encountered by migrant populations during their transit from their country of origin towards European destinations. This systematic review, therefore, aims to determine the health status and health risks faced by migrants during their journey to Europe. The specific objectives are to gather information on the characteristics of migratory paths and typical routes, to examine the experiences, risk factors and potentially traumatic events that occurred during the migration journey, to identify the health outcomes investigated, and to gather information on study designs and tools utilized, as well as the challenges faced by researchers in describing migration journeys and related outcomes. Additionally, our systematic review will focus on studies that formally assessed the association between experiences during transit and related health outcomes, to gain insight into the impact of these factors on the migrants’ health status recorded upon arrival in transit or destination countries.

## Materials and methods

### Search strategy and selection criteria

We conducted a systematic search following the Preferred Reporting Items for Systematic Reviews and Meta-Analyses 2020 (PRISMA) guidelines [20]. The study protocol was registered on PROSPERO (CRD42024513421). Following the PECO (Population, Exposure, Comparator, Outcome) framework (Table S1), we aimed to identify all available papers providing information on the health status of migrant populations (as defined by IOM) upon recent arrival in a transit or destination country in Europe (from less than 24 months), and on the characteristics or specific risk factors of their migration journey experience.

The search was conducted on January 5, 2024 over the following electronic databases: PubMed, Scopus, EMBASE. We used a comprehensive search string developed based on previous literature on the topic [21, 22], which included terms and synonyms related to migrant populations, health outcomes and European countries with spelling variations to ensure the capture of all relevant studies. Keywords referred to the population, exposure and outcome were searched in title/abstract, while keywords related to the setting were searched as free terms (all fields) to identify studies conducted in Europe, considering also the reported affiliations of the studies’ authors. The specific search strategy is listed in the Supplementary material (Table S2).

Studies were considered eligible for the systematic review if they met all the following criteria: a) focused on migrants’ health status upon arrival in a transit or destination country in Europe; b) the arrival in the country occurred less than 24 months prior to the study period (this period of time was selected to ensure that sufficient time was allocated for conducting the studies, recruiting participants in camps, and collecting data); c) provided information on the characteristics of migration journeys (at least one among duration, route and type of travel, transit countries) or on the specific risk factors experienced during the journey (experiences and events potentially harmful for health occurred during the journey, such as torture, sexual, physical and psychological violence); d) were published in English or Italian language; e) were published within the last 20 years. A 20-year time frame was selected to acquire the most up-to-date and reliable data related to migrant populations in Europe, considering the rapid changes in migration routes and policies over the years.

We excluded systematic or scoping reviews, commentaries, editorials, conference abstracts, case reports, and study protocols. Additionally, studies in which the origin of migrants was not specified, studies investigating the health status of migrants only before the start of their journey (in their country of origin), studies investigating the health status of foreign nationals residing in the destination country or individuals who have been in the destination country for more than 24 months, and studies providing information exclusively on subgroups of subjects presenting adverse health conditions, were excluded.

Title and abstract screening was performed using ASReview version 1.3, a software that uses Artificial Intelligence to speed up the title/abstract screening phase [23, 24]. ASReview uses active learning to influence the order of articles based on relevance for the inclusion process [25]. A single reviewer (IR) provided initial training data to ASReview and the classifier created a progressive ranking of the unseen records. The same reviewer then screened the relevant papers; when many articles are excluded in a row, it can be assumed that the articles listed after them can be labelled as irrelevant [26]. The software developers advise a screen-stop decision after 100–120 consecutively excluded studies. To ensure we would not miss relevant studies we decided on a screening-stop decision after 150 consecutively excluded studies [27, 28].

The full-text screening was then independently performed using Covidence software [29] by three reviewers (CC, CB and IR), considering all the papers included after the initial screening using ASReview. Any discrepancy was resolved through discussion. Once full texts were selected, reference lists were screened to search for potentially relevant studies.

### Data analysis

Data extraction was conducted by two different reviewers (IR and CB). For each included study, we extracted detailed information on four different topics: a) characteristics of the study (title, author, publication year, journal, study design, sources, period and language for data collection, who collected data, type of database, informed consent, European country in which the study was conducted); b) characteristics of the population (sample size, number of males/females, mean/median age, inclusion of children, accompanied/unaccompanied status, presence of vulnerability, level of education, occupation, terms used to define the migrant population, country/geographic area of origin, partnering status, legal status and planned steps; c) characteristics of exposure (time since arrival, reason for migration, travel duration, whether migration occurred by sea, air, or land, journey route, specific risk factors during journey, how they were measured, specific tools used, perpetrator of violence); d) characteristics of health outcomes (how they were measured, specific tools used, presence of formally assessed association, how they formally assessed the association).

Due to the heterogeneous characteristics of the included studies in terms of population, outcomes, and study design, it was not possible to conduct a meta-analysis. Results were qualitatively presented using graphical representations, creating geographic maps and heatmaps showing the association between exposures and outcomes investigated in the included studies, and the associations that were formally assessed in the included studies.

A risk of bias assessment was conducted for all included studies that formally investigated the association between exposures and outcomes, using the Joanna Briggs assessment tool for cross-sectional or cohort studies [30]. Two different reviewers (IR and CB) independently assessed the risk of bias for each item provided in the tool. In cases of disagreement, a third reviewer (CC) intervened to resolve the discrepancies. Following to the Joanna Briggs guidelines, instead of assigning a single overall score to each study, we provided a comprehensive assessment of all items. This approach allowed us to identify domains where studies were either lacking or adequate in terms of bias risk.

## Results

**Fig. 1:**
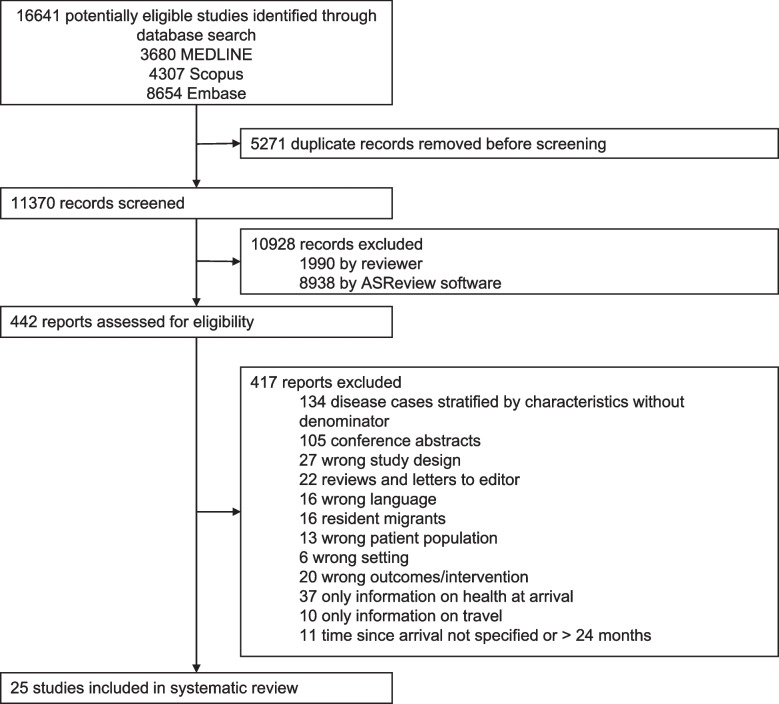
Study selection

After removing duplicates, we identified a total of 11,370 records across the three databases, of which 78.6% were marked as not relevant after employing the ASReview tool. In the full-text screening phase, 442 papers were assessed for eligibility, and ultimately, 25 papers were included in the review (Fig. 1).

### Studies’ characteristics

Table 1 and Table S3 present the general characteristics of the included studies. All studies were published from 2017, with the majority from 2020 (*n* = 16, 64.0%). The cross-sectional study design was the most adopted, investigating characteristics of exposures and outcomes of interest simultaneously (*n* = 21, 84.0%). Four studies (16.0%) employed a longitudinal design, investigating migrants’ characteristics during the transit phase and after their resettlement in destination countries [31–34]. In most cases (*n* = 19, 76.0%) researchers collected quantitative information. Only one study was qualitative (4.0%) [35], while mixed methods research was employed in the remaining five studies (20.0%).

**Table 1:** Characteristics of included studies

Author, publication year	Study design	Data collection	Period for data collection	Organization(s) involved	Sample size (% males)	Age	Study location	Country/geographic area of origin
Arsenijević et al. (2017) [27]	CS, mixed methods	PB	2015–2016	MSF	992 (69∙5%)	5–17y: 12% (29% UM); 18–64y: 86%; ≥ 65y: 2%	Serbia	Syria (46%), Afghanistan (26%), Iraq (9%), Morocco (5%), Iran (4%), others (10%)
Arsenijević et al. (2018) [28]	CS, qualitative	O	2017	MSF	30 (100∙0%)	Mean 23∙3y (25% UM)	Serbia	Afghanistan (63%), Algeria (10%), Iraq (10%), others (17%)
**Ben Farhat et al. (2018)** [29]	CS, mixed methods	PB	2016–2017	MSF	728 (59∙3%)	Median 18y (IQR 7–30y); < 15y: 43∙4%	Greece	Syria (100%)
Bergevin et al. (2021) [30]	CS, quantitative	PB	2017–2018	Association des Juniors en Pédiatrie, France Terre d'Asile	107 (88∙8%)	Median 15y (IQR 14 − 16y); 100∙0% UM	France	Sub-Saharan Africa (63%), Northern Africa (10%), Afghanistan-Pakistan-Bangladesh (20%)
Bouhenia et al. (2017) [31]	CS, quantitative	PB	2015	MSF	425 (0.5%)	Median 25y (IQR 21–30y); 4% < 15y	France	Sudan (33∙3%), Afghanistan (18∙3%), Iraq (12.8%), Iran (11∙5%), Syria (7∙3%), Eritrea (6∙9%), Pakistan (3∙0%), others (6∙0%)
Chernet et al. (2021) [32]	L, quantitative	PB	2016	Swiss Tropical and Public Health Institute	107 (88∙8%)	Median 25y (IQR 21–29y)	Switzerland	Eritrea (100∙0%)
Crepet et al. (2017) [33]	CS, quantitative	PB	2014–2015	MSF, Italian Ministry of Health, Medici per i Diritti Umani, UNHCR, Save the Children, IOM	385 (91∙2%)	Median 23y (IQR 20–27y)	Italy	Nigeria (20%), Gambia (17%), Senegal (13%), Mali (21%), Bangladesh (11%), other West African (15%), others (11%), unknown (1%)
**Fontanelli Sulekova et al. (2019)** [34]	CS, quantitative	PB	2017	Sanitary Bureau of Asylum Seekers Center, Auxilium Società Cooperativa Sociale	364 (77∙7%)	Median 23y (range 4–47y for F, range 1–76y for M); 0∙3% < 15y	Italy	Western Africa (41∙7%), Eastern Africa (38∙5%), Middle Africa (2∙2%), North Africa (7∙7%), West Asia (3%), Southern Asia (6∙9%)
Garoff et al. (2021) [35]	CS, quantitative	PB	2018	Finnish Institute for Health and Welfare, Finnish Immigration Service	784 (60∙3%)	18–29y: 37∙4%; 30–39y: 37∙1%; over 39y: 25∙5%	Finland	Russia (29∙2%), Middle East and North Africa (43∙6%), other regions of Africa (16∙5%), others
Giannopoulou et al. (2022) [36]	CS, quantitative	O	2018	National and Kapodistrian University of Athens, Attikon University Hospital	90 (84∙4%)	Mean 16∙2 y (SD 1∙2y); 100∙0% UM	Greece	Syria (47∙8%), Afghanistan (14∙4%), Pakistan (10%), Iran (7∙8%), Iraq (5∙6%), North Africa (7∙8%), other African countries (3∙3%)
Giordano et al. (2019) [37]	CS, quantitative	O	2014	NGOs: Albero della Vita Foundation, Share With All People	271 (59∙0%)	Mean 10y (SD 2∙11y), range 6-14y; 100∙0% UM	Italy	Syria (80%), Palestine (20%)
**Guarch-Rubio et al. (2020)** [38]	CS, quantitative	O	2019	University of Madrid and Limerick	54 (94∙4%)	18-25y: 53∙7%; 26-34y: 40∙7%; 35 + y: 5∙5%	Bosnia-Herzegovina	Algeria (38∙9%), Morocco (18∙5%), Iraq (14∙8%), Syria (14∙8%), Palestine (3∙7%), Tunisia (3∙7%), others (5∙7%)
**Haj-Younes et al. (2020)** [39]	L, quantitative	PB	2017–2018	The Research Council of Norway, IOM	353 (48∙4%)	Median 34y; (IQR 27–41y)	Lebanon—Norway	Syria (100∙0%)
**Jankovic-Rankovic et al. (2022)** [40]	CS, mixed methods	O	2018	University of Notre Dame, Commissariat for Refugees and Migration of Serbia	76 (53∙9%)	Mean 30∙1y (SD 7∙7y); range 18-50y	Serbia	Afghanistan (48∙7%), Iran (28∙9%), Syria (3∙9%), Pakistan (2∙6%), Iraq (2∙6%)
**Jankovic-Rankovic et al. (2020)** [41]	CS, mixed methods	O	2017–2018	University of Notre Dame, Commissariat for Refugees and Migration of Serbia	111 (64∙9%)	Mean 28∙5y (SD 8∙5y); range 18–55y	Serbia	Afghanistan (61∙1%), Pakistan (5∙6%), Iraq (8∙3%), Iran (8∙3%)
**Prestileo et al. (2022)** [42]	CS, quantitative	PB	2015–2017	Take Care Advocacy (ITaCA), Healthcare Service and Hospital of Palermo	1911 (67∙8%)	Mean 24y, range 14-41y; 38% < 18y	Italy	Gambia (16∙3%), Nigeria (15∙8%), Senegal (9∙7%), Ivory Coast (8∙6%), Ghana (7∙6%), Guinea (7∙5%), Mali (7∙2%), Sudan (3∙3%)
**Purić et al. (2019)** [43]	CS, quantitative	O	2014	UNHCR Serbia	226 (88∙0%)	Mean 27∙35 y (SD = 8∙45y)	Serbia	Syria (45%), Afghanistan (15%), Somalia (17%)
**Rodolico et al. (2020)** [44]	CS, quantitative	PB	2016–2017	Italian Red Cross, University Hospital of Catania, Ministry of Internal Affairs	98 (75∙5%)	Median 23y (IQR: 20-27y)	Italy	Nigeria (32∙6%), Senegal (14∙3%), Gambia (9∙2%), Ghana (8∙2%), Mali (8∙2%), Ivory Coast (7∙1%), Eritrea (4∙1%)
Segneri et al. (2022) [45]	CS, mixed methods	O	2015	INMP	112 (73∙2%)	Mean 24∙4y (SD 6∙7y); range 10–46	Italy	Eritrea (24∙1%), Nigeria (23∙2%), Sudan (8∙0%), Gambia (7∙1%), Ghana (5∙4%), Syria (6∙3%)
**Strømme et al. (2020)** [46]	CS, quantitative	PB	2017–2018	Regional Committee for Research Ethics of Norway, Department of Global Public Health and Primary Care, University of Bergen	827 (58∙9%)	Mean 33y (SD 10y)	Lebanon—Norway	Syria (100∙0%)
**Strømme et al. (2020)** [47]	L, quantitative	PB	2017–2018	University of Bergen, Norwegian Institute of Public Health, Regional Committee for Research Ethics of Norway, IOM	353 (48∙4%)	Median 34y (IQR 27-41y)	Lebanon—Norway	Syria (100∙0%)
Bronsino et al. (2020) [48]	CS, quantitative	Secondary data	2016–2017	Italian Red Cross, University of Turin	2484 (0∙0%)	< 18y: 10∙3%, 18-24y: 55∙5%, 25-34y: 27∙7%, > 35 y: 6∙5%	Italy	Sub Saharan Africa (67∙9%), Horn of Africa (22∙5%), Middle East (3∙6%), North Africa (3∙8%), Central-South Africa (1∙7%)
**Poole et al. (2018)** [49]	CS, quantitative	PB	2017	IOM, Harvard TH Chan School of Public Health	135 (59∙2%)	Median 30y (IQR 24–37y)	Greece	Syria (88∙9%)
**Strømme et al. (2021)** [50]	L, quantitative	PB	2017–2018	University of Bergen, IOM	353 (48∙4%)	Median 34y (IQR 27–41y)	Lebanon—Norway	Syria (100∙0%)
**Vukčević Marković et al. (2023)** [51]	CS, quantitative	O	2021	University (Department of Psychology)	201 (89∙0%)	Mean 28∙7y (SD 8∙8y), range 14–65; 2∙9% < 18y	Serbia	Syria (24∙4%), Afghanistan (19∙7%), Morocco (17∙1%), Iran (8∙3%), Iraq (5∙2%), Bangladesh (4∙7%), others

In bold, we identified the studies that formally assess the association between investigated exposures and outcomes

*CS* cross-sectional, *L* longitudinal, *PB* population-based, *O* opportunistic sample, *MSF* Médecins sans Frontierès, *IOM* International Organization for Migration, *INMP* National Institute for Health, Migration and Poverty, *UM* unaccompanied minors, *y* years, *SD* standard deviation, *IQR* interquartile range

The majority of the studies employed population-based samples, enrolling all individuals residing in camps and dedicated facilities during the study periods, while in other cases (*n* = 9, 36.0%) the investigators employed opportunistic samples. One study (4.0%) used secondary data, relying on information from medical records and health documentation [36]. The target population participated in the studies mainly while hosted in refugee camps/asylum centers (*n* = 15, 60.0%), or in facilities where migrants admitted for resettlement in Europe attended mandatory pre-departure educational activities (*n* = 4, 16.0%).

We observed that Higher Education Institutes (HEIs) were the most frequently involved organizations (*n* = 6, 24.0%) in the implementation of the studies. In other instances, HEIs were responsible for the studies with the support of IOM (*n* = 4, 16.0%). Médecins sans Frontierès (MSF) was the non-governmental organization (NGO) most frequently involved (present in four out of six studies carried out by NGOs). Public Health Institutes (such as the Swiss Tropical and Public Health Institute, the Finnish Institute for Health, the National Institute for Health, Migration and Poverty) were responsible for four (16.0%) of the included studies. In four studies (16.0%), multiple organisations were involved and collaborated in the study process.

In the included studies, data were collected over limited time periods, with mean durations of seven months. The data collection period ranged from a minimum of 15 days to a maximum of 26 months. In most instances, data collection was carried out by the study researchers with the assistance of cultural mediators (*n* = 7, 28.0%), healthcare workers with mediators (*n* = 6, 24.0%), or by researchers or healthcare workers alone (*n* = 10, 40.0%). The healthcare workers involved included physicians, nurses, psychologists, and others.

### Populations’ characteristics

Table 1, Table S3 and Table S4 contain information regarding the populations’ characteristics. The sample sizes were relatively small, ranging from 30 to 2484, with a mean of 463 subjects. The distribution of males and females was homogeneous. Overall, 14 studies (56.0%) were conducted on young adults only, while 11 (44.0%) included also accompanied or unaccompanied minors. Among these, three studies (12.0%) were conducted on minors only, comprising a population aged between 6 and 17 years old [37–39]. Almost all studies defined their population as either refugees or asylum seekers. Although these terms are distinct, they were used interchangeably by study researchers to refer to study participants regardless of their legal status at the time of the research.

**Fig. 2:**
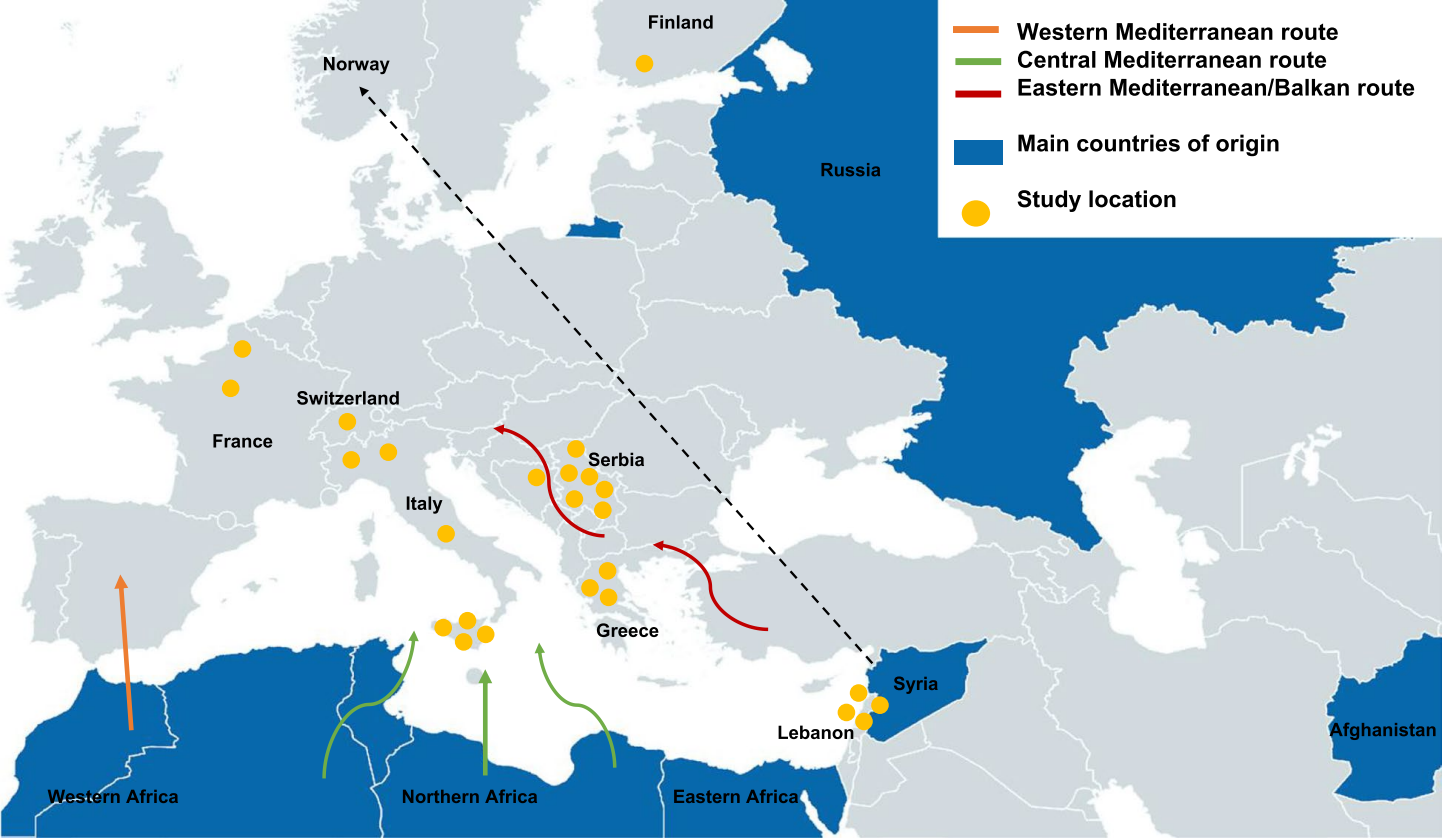
Study locations, main countries of origin and migratory routes towards Europe. The black arrow represents four longitudinal studies in which the initial phases were conducted in Lebanon, and follow-up was conducted in Norway

Figure 2 presents a comprehensive overview of the study locations and the main countries of origin of the included populations. Most of the studies involved populations originating from Syria (*n* = 11, 44.0%). Ten studies (40.0%) included populations from the African continent, especially Western Africa (Gambia, Senegal, Nigeria, Guinea, Mali). In other studies, the main country of origin was Afghanistan (*n* = 3, 12.0%), while one study included populations mainly originating from Middle Eastern or North African countries [40].

Regarding the location of the studies, the majority were conducted in Italy and Serbia (*n* = 7, 28.0% and *n* = 6, 24.0%, respectively), followed by Norway (*n* = 4, 16.0%). The remaining studies were conducted in Greece (*n* = 3, 12.0%), France (*n* = 2, 8.0%), Switzerland (*n* = 1, 4.0%), Bosnia-Herzegovina (*n* = 1, 4.0%) and Finland (*n* = 1, 4.0%). Not all studies provided information on the migratory routes used by migrants but based on countries/areas of origin of included populations and study locations we can infer two primary paths: the Central Mediterranean route, to reach Italy, France and Switzerland coming from Western, Northern and Eastern Africa, and the Balkan route, with migrants originating mainly from Syria, Afghanistan and Iraq. It is noteworthy that we did not identify any study on migrants travelling via the Western Mediterranean route.

### Journey characteristics, specific risk factors experienced during the journey and health outcomes

The migration journey experience was divided into two categories: journey characteristics (*n* = 24 studies, 96.0%) and specific risk factors experienced during travel (*n* = 21 studies, 84.0%) (Table S3). A total of 19 studies (76.0%) reported information on the time since arrival in the country where the study was conducted, expressed in median/mean number of days/months/years. In the included studies, migrants had been in those countries for a few days to several months, and in some cases, for more than a year. Two studies (8.0%) stated that migrants were newly arrived without providing further details [40, 41]. A total of five studies (20.0%) provided detailed information on the reasons behind migration, which was predominantly driven by factors such as war and conflict, the pursuit of international protection, insecurity, political instability and persecution (Table S3). The duration of travel was reported in 19 studies (76.0%), either as the median time of travel duration (and I-III quartile) or using specific categories. In the included studies, the journeys lasted from one to 60 months (see Additional file 2). Only eight studies (32.0%) specified the journey type, indicating whether migration occurred by sea, air, or land. Additionally, data on the presence of a transit country, the number of transit countries, the median duration of stay in transit countries, and the transit countries crossed were obtained from 11 out of 25 studies (44.0%) (Table S3).

Specific risk factors pertaining to the transit phase included, among others, violent events and trauma experiences, potentially traumatic events, and difficulties encountered during the journey. These risk factors were presented in the included studies either by considering the total number of events experienced by the subjects or by providing detailed information regarding the type of events (torture, threats, sexual or physical violence, robbery, incarceration, forced labor and others). While journey characteristics were consistently assessed through specifically developed questions, validated questionnaires were utilized for evaluating the risk factors experienced during travel. These instruments were either specifically designed for journey experiences or adapted from more generic questionnaires on trauma [42] (Table S3).

**Fig. 3:**
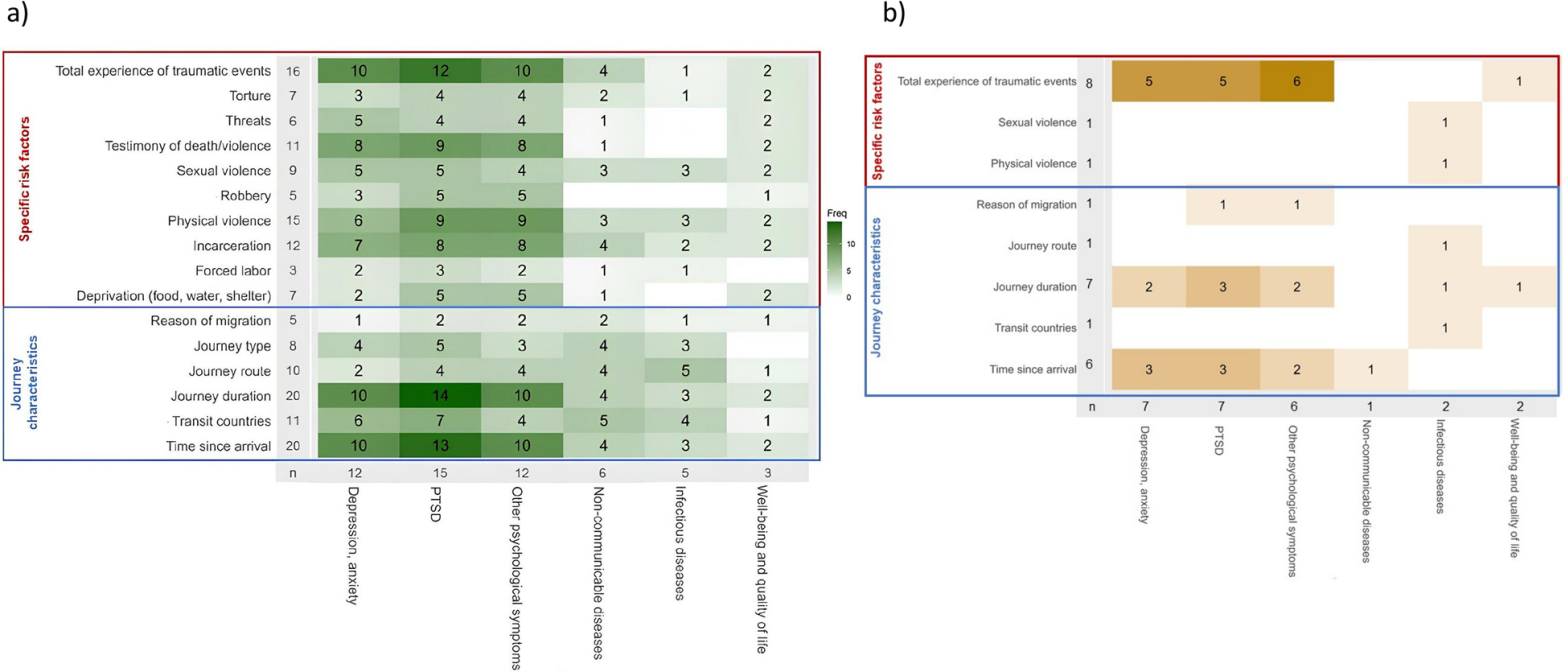
Heatmap for combinations of investigated exposures and outcomes (**a**) and associations between investigated exposures and outcomes (**b**)

Most of the included studies focused on mental health (*n* = 15, 60.0%) as the main health outcome of interest, investigating the presence of anxiety and depression disorders, post-traumatic stress disorder (PTSD) and other psychological symptoms. Studies on physical health (*n* = 4, 16.0%) examined the presence of non-communicable diseases (NCDs) and infectious diseases. One study was exclusively focused on perceived health, including well-being and perceived quality of life in participants (*n* = 1, 4.0%) [34]. Five studies (20.0%) addressed multiple health outcomes (Fig. 3a). The following heatmap (Fig. 3a) describes all potential combinations of exposures and outcomes investigated in the included papers. Exposures were categorised into specific risk factors experienced during the journey and journey characteristics. Health outcomes were classified into mental health, including anxiety, depression, other psychological symptoms and PTSD symptoms, non-communicable diseases, infectious diseases, well-being and quality of life.

For each cell, we reported the total number of studies providing information for that combination of exposure and outcome. The colour intensity in each cell reflects the number of studies, indicating that the majority of the available literature focused on mental health problems in combination with the total number of traumatic events experienced, the duration of the journey, and the time since arrival in the countries where the studies were conducted. As previously stated, only 14 out of 25 studies (56.0%) formally assessed the associations between exposures and health outcomes [31–34, 42–51]. The associations evaluated in these studies are presented in the heatmap (Fig. 3b). Most of these studies focused on the relationship between mental health problems and the total number of traumatic events, showing that increased exposure to various types of traumatic experiences during the journey was associated with higher risks of experiencing anxiety, depression and PTSD [31, 42, 44, 51]. Furthermore, a higher risk of mental health problems was positively linked to the length of the journey and the time since arrival [45, 46, 49, 50]. In addition, a few studies also examined the positive association between exposure to sexual and physical violence, the selection of specific journey routes, and the occurrence of infectious diseases [43, 47].

With regard to the characteristics evaluated in the risk of bias assessment (Table S5), it was observed that nearly all included studies adequately defined their inclusion criteria and described subjects and study settings. Furthermore, all studies employed valid methodologies for measuring exposure and outcomes, utilising validated questionnaires or surveys specifically developed for the study’s purposes. However, it is noteworthy that while all studies identified possible confounding factors, some of them (*n* = 4, 16.0%) did not employ specific strategies to address these confounders in the statistical analysis. This may be attributed to the limited sample sizes available in the included studies.

## Discussion

We conducted a systematic review of published studies investigating the health status of migrant populations upon their arrival in European countries, with a focus on the experiences and risk factors associated with the migration journey. A total of 25 studies met the inclusion criteria, encompassing various study designs, populations, and health outcomes. Of these, only 14 formally assessed the association between transit-related exposures and health outcomes.

In the studies included in the review, mental health disorders and psychological trauma emerged as the predominant health issues investigated among migrants. Several studies have reported high prevalence rates of PTSD, depression, and anxiety among migrant populations, highlighting the urgent need for mental health support upon arrival [48, 49, 52]. The risk of infectious diseases was another key health outcome identified. Studies have highlighted the prevalence of conditions such as tuberculosis, hepatitis, and various parasitic infections, which are exacerbated by poor living conditions and limited access to healthcare during transit [37, 43]. Sexually transmitted diseases (STDs) were also reported among the prevalent infectious diseases identified [47]. Chronic NCDs have been less studied in the included papers although they represent a cause for concern, due to the significant burden they may pose to healthcare systems in receiving countries [33]. Indeed, the available studies report that migrants are at high risk of NCDs, particularly cardiovascular, musculoskeletal and respiratory diseases, for which they often do not receive adequate treatment [31, 36].

The systematic review highlights that migrants in transit may be exposed to various risk factors, encompassing a range of traumatic events, including physical and verbal violence, torture and threats, sexual violence and exploitation, detention, robbery and forced labour. These events can occur in many ways; migrants may experience them personally or witness them [53, 54]. Exposure to these risks may also be gender-related, as women and girls may be more likely to experience sexual violence, while men and boys may also endure physical violence, forced labour, and inhuman and degrading treatments [55–57]. However, sexual violence against migrant boys and men is often underreported due to social and cultural stigma, as well as the belief that men cannot be victims of rape [58]. The impact of experiencing these traumatic events can be multifaceted, with potential health effects ranging from physical (such as wounds, fractures, and infectious diseases) to mental and psychological (such as anxiety, depression, PTSD, and substance abuse). Researchers have highlighted significant challenges in documenting individual histories, particularly when reporting instances of sexual violence [49].

Most of the studies in our systematic review related traumatic events to anxiety and depression symptoms, PTSD, and other mental disorders [42, 44–46, 51], with comparatively less research on their effect on physical health [47]. The prevalence and type of violent and traumatic experiences that occurred during transit and pushbacks may influence both the incidence and severity of mental disorders, which may also become chronic. Indeed, in host countries, many migrants either lack access to mental health services or encounter barriers to obtaining them, facing interruptions in continuous care [59]. Even if less investigated in the included studies, communicable diseases remain a major health issue for migrating populations. Sexually transmitted diseases are closely linked to sexual violence, which may occur during transit and displacement in dangerous transit countries [36, 47, 56].

The main migration routes to Europe comprise the Western, Central, and Eastern Mediterranean, which include maritime pathways, along with the Balkan route, used predominantly by migrants traveling by land [7]. The real impact of the Mediterranean routes on migrant populations’ health is difficult to estimate, as the total number of people drowning during the sea crossing is unknown [60]. Different routes are chosen based on individual sociodemographic variability and journey-related factors, with both elements contributing to shaping dynamic migration trajectories [61].

A few studies examined the role of the characteristics of the migratory route, the journey duration, transit countries, and countries of origin in impacting health outcomes. These aspects tend to determine the violence and events experienced by migrants during transit; for instance, routes crossing countries with notorious exploitation systems for migrant populations, considerably increase the risk of experiencing physical violence, sexual exploitation, rape, torture, and incarceration, with negative effects on psychological health [44, 56]. Regional differences in the types of physical violence experienced are notable, with people transiting through countries in the WHO African and Eastern Mediterranean regions frequently reporting detention and torture, often as a result of political violence or actions by state actors [11]. Moreover, violence can also be encountered in the receiving country, compounding the trauma experienced during transit. Additionally, the practice of pushbacks, which involve forcibly returning migrants to their country of origin or a third country, significantly worsens health outcomes [51, 62].

In some studies, researchers have focused on the link between the length of the journey and migrants’ mental health [45, 46]. Results suggest that prolonged stays in refugee camps during the asylum process, as well as dangerous and protracted long journeys, predispose individuals to extreme physical and psychological distress, heightening the risk of depression [50]. Conversely, shorter stays in transit countries before resettlement were associated with better mental health outcomes over time [33]. Moreover, chronic conditions could suffer from extreme delays in diagnosis and treatment due to the journey itself and lack of access to healthcare in the receiving countries, while traveling in small clusters of people, with prolonged sharing of time and places, may influence the prevalence of parasitic infestations [43].

In studies with a longitudinal design, most migration-related stressors were found to be more closely associated with chronic pain and mental health problems after resettlement compared to the transit phase [32–34, 41]. Researchers delved into the concept of general well-being and self-reported health for these populations, underlining how mental health outcomes improved in the early post-migration phase [33] along with the quality of life over time [34]. These studies provide useful insight into the sequential changes in health among people moving from completely disparate settings, but as reported in the review, their number is limited.

The main strengths of our analysis lie in the comprehensive search string used and in the specific definition of our inclusion and exclusion criteria. Our criteria ensured the retrieval of papers that effectively presented information regarding both exposures and health outcomes related to the transit phase, while excluding a significant number of publications focused solely on the characteristics of the journey or on health upon arrival in destination countries. The limitations of our review are primarily related to the scarcity of available research on the topic and to the heterogeneous characteristics of the included studies, which did not allow us to conduct a meta-analysis of the results.

To our knowledge, various additional health outcomes have not been adequately considered in this research field thus far, including the health of vulnerable populations such as pregnant women and people with disabilities. Conversely, while certain topics, such as the prevalence of NCDs or STDs, have been widely explored in studies conducted on migrant populations upon arrival in destination countries in Europe [16], there may still be room for an increase in the number of studies reporting also information on the migration journey as possible risk factor. Interestingly, we did not encounter papers that investigated the characteristics of migrants following the Eastern Mediterranean route, while others, especially the Balkan route, were well represented. This finding underscores the presence of gaps in the evaluation of migration trajectories in the European context and suggests the need for further exploration in future research. Filling these gaps could enhance our understanding of the challenges posed by the transit phase.

The results highlighted by this systematic review underscore the importance of prioritizing the unique health needs of these populations [63], considering the possible role of several different determinants and experiences in influencing individual well-being. In particular, we suggest that future studies should pay greater attention to the potential impact of the transit phase on the health profile of these populations, in conjunction with other factors pertaining to the pre-migration and post-migration phases [64, 65].

In their countries of origin, migrants are forcibly exposed to war, conflicts, persecution, poverty, and hunger. Upon arriving in their destination country, migrants can still face barriers in accessing healthcare and utilizing screening services [64]. Various studies have explored the role of these determinants [16], yet research on the threats posed during the transit phase remains scarce, fragmented, and only emerged in very recent years. During the migration journey, migrants are exposed to secondary trauma, including physical safety risks, kidnapping, imprisonment, lack of food and water, and difficult living conditions in refugee camps [3]. These conditions, combined with the absence of structured and organized flows of medical records [19], make it methodologically difficult to realize and conduct observational studies, thus influencing the amount of information available to researchers.

## Conclusion

During travel, as well as in the countries of transit, migrants’ health outcomes will depend on the travel conditions and mode of travel, including the number and type of potentially traumatic experiences. The qualitative results provided by our systematic review highlight the scarcity of studies that offer insight into migrants’ journeys, their characteristics, and the specific risk factors experienced in relation to health outcomes. The number of studies investigating both the journeys’ characteristics and related health outcomes is low, and the number of studies that formally assessed the presence of an association between the experiences of the transit phase and health outcomes is even lower.

Our findings underscore the limitations of our knowledge regarding the determinants of the health of migrant populations, including migration processes among them. The lack of information in our possession regarding the difficulties experienced during migration journeys and related traumatic events limits the discussion on the impact of the transit phase on migrants’ health.

## Supplementary Information

Additional file 1: Table S1: PECO framework; Table S2: Search strings for included databases; Table S4: Studied populations’ characteristics; Table S5: Risk of bias table for studies that formally investigated the association between exposures and outcomes.

Additional file 2: Table S3: General characteristics of included studies.

## Data Availability

All data generated or analysed during this study are included in this published article and its supplementary information files. Data availability No datasets were generated or analysed during the current study.

## Abbreviations

WHO: World Health Organization
IOM: International Organization for Migration
MSF: Médecins sans Frontierès
NGO: Non-governmental organization
PTSD: Post-traumatic stress disorder
NCDs: Non-communicable diseases
STDs: Sexually transmitted diseases
