# Supplementary material for: Assessing the health status of migrants upon arrival in Europe: a systematic review of the adverse impact of migration journeys
Source: Global Health. 2024 Sep 27;20:69. doi: 10.1186/s12992-024-01075-3 (PMC11438409; doi:10.1186/s12992-024-01075-3)
Supplement: Supplementary file 1 — Additional file 1: Table S1: PECO framework; Table S2: Search strings for included databases; Table S4: Studied populations’ characteristics; Table S5: Risk of bias table for studies that formally investigated the association between exposures and outcomes. [file 12992_2024_1075_MOESM1_ESM.docx]

**Supplementary materials**

**Table S1: PECO framework.**

| **Population** | Migrants in transit arriving in countries in Europe, whether as a final or intermediate destination (for less than 24 months) |
| --- | --- |
| **Exposure** | Journey experience through the country of origin towards the country of destination in Europe |
| **Comparator** | None |
| **Outcome** | Health status recorded at arrival in a transit country in Europe or a destination country in Europe (from less than 24 months) |
| **Setting** | European countries |

**Table S2: Search strings for included databases.**

| **PubMed**  (migrant*[Title/Abstract] OR emigrant*[Title/Abstract] OR immigrant*[Title/Abstract] OR migrat*[Title/Abstract] OR emigrat*[Title/Abstract] OR refugee*[Title/Abstract] OR "asylum seeker*"[Title/Abstract] OR "displaced population*"[Title/Abstract] OR "displaced people"[Title/Abstract] OR immigrat*[Title/Abstract] OR foreigner*[Title/Abstract] OR "transients and migrants"[MeSH Terms] OR "emigrants and immigrants"[MeSH Terms]) AND (journey*[Title/Abstract] OR mobility[Title/Abstract] OR crossing[Title/Abstract] OR transit[Title/Abstract] OR travel*[Title/Abstract] OR moving[Title/Abstract] OR route*[Title/Abstract] OR border*[Title/Abstract] OR itinerary[Title/Abstract] OR itinerant[Title/Abstract] OR passage*[Title/Abstract] OR corridor*[Title/Abstract]) AND (abuse*[Title/Abstract] OR addiction*[Title/Abstract] OR assault*[Title/Abstract] OR death*[Title/Abstract] OR disease*[Title/Abstract] OR disorder*[Title/Abstract] OR illness*[Title/Abstract] OR infection*[Title/Abstract] OR injur*[Title/Abstract] OR mortality[Title/Abstract] OR morbidity[Title/Abstract] OR rape[Title/Abstract] OR violence[Title/Abstract] OR wellbeing[Title/Abstract] OR well-being[Title/Abstract] OR "health problem*"[Title/Abstract] OR "health outcome*"[Title/Abstract] OR "health difficult*"[Title/Abstract] OR "health complication*"[Title/Abstract] OR "health status"[Title/Abstract] OR "health challenge*"[Title/Abstract] OR suicide*[Title/Abstract] OR murder* [Title/Abstract] OR homicide[Title/Abstract] OR "gun wound*"[Title/Abstract] OR beatings[Title/Abstract] OR slavery[Title/Abstract] OR starvation[Title/Abstract] OR isolation[Title/Abstract] OR "stab wound*"[Title/Abstract] OR torture*[Title/Abstract] OR drowning[Title/Abstract] OR burn*[Title/Abstract]) AND (Europe OR "European Union" OR "Eastern Europe" OR EU OR "European Free Trade Association" OR EFTA OR Euro* OR Austria OR Belgium OR Bulgaria OR Croatia OR Cyprus OR "Czech Republic" OR Denmark OR Estonia OR Finland OR France OR Germany OR Greece OR Hungary OR Ireland OR Italy OR Latvia OR Liechtenstein OR Lithuania OR Luxembourg OR Malta OR Netherlands OR Norway OR Poland OR Portugal OR Romania OR Slovakia OR Slovenia OR Spain OR Sweden OR Switzerland OR "United Kingdom" OR UK OR England OR Scotland OR Wales OR "Northern Ireland") Filters: from 2003 - 2024  **Total: 3680** |
| --- |
| **Scopus**  TITLE-ABS ( migrant* OR emigrant* OR immigrant* OR migrat* OR emigrat* OR refugee* OR "asylum seeker*" OR "displaced population*" OR "displaced people" OR immigrat* OR foreigner* ) AND TITLE-ABS ( journey* OR mobility OR crossing OR transit OR travel* OR moving OR route* OR border* OR itinerary OR itinerant OR passage* OR corridor* ) AND TITLE-ABS ( abuse* OR addiction* OR assault* OR death* OR disease* OR disorder* OR illness* OR infection* OR injur* OR mortality OR morbidity OR rape OR violence OR wellbeing OR well-being OR "health problem*" OR "health outcome*" OR "health difficult*" OR "health complication*" OR "health status" OR "health challenge*" OR experience* OR suicide* OR murder* OR homicide OR "gun wound*" OR beatings OR slavery OR starvation OR isolation OR "stab wound*" OR torture* OR drowning OR burn* ) AND ( TITLE-ABS-KEY-AUTH ( europe OR "European Union" OR "Eastern Europe" OR eu OR "European Free Trade Association" OR efta OR euro* OR austria OR belgium OR bulgaria OR croatia OR cyprus OR "Czech Republic" OR denmark OR estonia OR finland OR france OR germany OR greece OR hungary OR ireland OR italy OR latvia OR liechtenstein OR lithuania OR luxembourg OR malta OR netherlands OR norway OR poland OR portugal OR romania OR slovakia OR slovenia OR spain OR sweden OR switzerland OR "United Kingdom" OR uk OR england OR scotland OR wales OR "Northern Ireland" ) ) AND PUBYEAR > 2002 AND PUBYEAR < 2025  **Total: 4307** |
| **Embase**  ((migrant* or emigrant* or immigrant* or migrat* or emigrat* or refugee* or "asylum seeker*" or "displaced population*" or "displaced people" or immigrat* or foreigner*) and (journey* or mobility or crossing or transit or travel* or moving or route* or border* or itinerary or itinerant or passage* or corridor*) and (abuse* or addiction* or assault* or death* or disease* or disorder* or illness* or infection* or injur* or mortality or morbidity or rape or violence or wellbeing or well-being or "health problem*" or "health outcome*" or "health difficult*" or "health complication*" or "health status" or "health challenge*” or suicide* or murder* or homicide or "gun wound*" or beatings or slavery or starvation or isolation or "stab wound*" or torture* or drowning or burn*)).ab,ti. and (Europe or "European Union" or "Eastern Europe" or EU or "European Free Trade Association" or EFTA or Euro* or Austria or Belgium or Bulgaria or Croatia or Cyprus or "Czech Republic" or Denmark or Estonia or Finland or France or Germany or Greece or Hungary or Ireland or Italy or Latvia or Liechtenstein or Lithuania or Luxembourg or Malta or Netherlands or Norway or Poland or Portugal or Romania or Slovakia or Slovenia or Spain or Sweden or Switzerland or "United Kingdom" or UK or England or Scotland or Wales or "Northern Ireland").af.  **Total: 8654** |

**Table S4: Studied populations’ characteristics.**

| **Populations’ characteristics (n=25)** | |
| --- | --- |
| **Sample size** | |
| **Total**  Mean (SD)  Median (I-IIIQ)  Min-max | 46.1 (590.4)  271 (107-425)  30-2484 |
| **Males**  Mean (SD)  Median (I-IIIQ)  Min-max | 231.2 (282.0)  160 (74-283)  0-1296 |
| **Females**  Mean (SD)  Median (I-IIIQ)  Min-max | 231.5 (495.4)  55 (22-296)  0-2484 |
| **Definition of migrants** | |
| Refugees  Asylum seekers  Migrants  More than one of the above | 11 (44.0%)  7 (28.0%)  3 (12.0%)  4 (16.0%) |
| **Main country of origin** | |
| Syria  Afghanistan  Africa  Middle East and North Africa | 11 (44.0%)  3 (12.0%)  10 (40.0%)  1 (4.0%) |
| **Main country of origin = Africa (n=10)**  Western Africa (Gambia, Senegal, Nigeria, Guinea, Mali)  Eastern Africa (Eritrea, Somalia)  Northern Africa (Sudan, Algeria, Morocco)  Sub-saharan Africa | 4 (40.0%)  2 (20.0%)  2 (20.0%)  2 (20.0%) |

**Table S5: Risk of bias table for studies that formally investigated the association between exposures and outcomes.**

| **Cross-sectional studies** | | | | | | | | | |
| --- | --- | --- | --- | --- | --- | --- | --- | --- | --- |
|  | Criteria for inclusion specified | Subjects and setting described | Exposure measured in a valid way | Objective criteria for migrant status | Confounding factors identified | Strategies to deal with confounding factors stated | Outcomes measured in a valid way | Appropriate statistical analysis |  |
| Ben Farhat, 2018 | y | y | y | y | y | y | y | y |  |
| Fontanelli Sulekova, 2019 | y | y | y | y | y | y | y | y |  |
| Guarch-Rubio, 2020 | y | y | y | y | y | n | y | y |  |
| Jankovic-Rankovic, 2022 | n | y | y | y | y | Y | y | y |  |
| Jankovic-Rankovic, 2020 | y | y | y | y | y | Y | y | y |  |
| Prestileo, 2022 | y | y | y | y | y | Y | y | y |  |
| Purić, 2019 | y | y | y | y | y | n | y | y |  |
| Rodolico, 2020 | y | y | y | y | y | Y | y | y |  |
| Strømme, 2020 | y | y | y | y | y | Y | y | y |  |
| Poole, 2018 | y | y | y | y | y | Y | y | y |  |
| Vukčević Marković, 2023 | y | y | y | y | y | n | y | y |  |

| **Longitudinal studies** | | | | | | | | | | | |
| --- | --- | --- | --- | --- | --- | --- | --- | --- | --- | --- | --- |
|  | Groups recruited from the same population | Exposures measured similarly for exposed and unexposed | Exposure measured in a valid way | Confounding factors identified | Strategies to deal with confounding factors stated | Participants free of the outcome at the start or at exposure | Outcomes measured in a valid way | Follow up time reported and sufficient for outcomes | Follow up complete (or reasons to loss) | Strategies to address incomplete follow up | Appropriate statistical analysis |
| Chernet 2021 | NA | NA | y | y | n | NA | y | y | y | y | y |
| Strømme, 2021 | NA | NA | y | y | y | NA | y | y | y | y | y |
| Strømme, 2020 | NA | NA | y | y | y | NA | y | y | y | y | y |
| Haj-Younes, 2020 | NA | NA | y | y | y | NA | y | y | y | y | y |

Notes: y = yes; n = no; NC = not clear; NA = not applicable.

Following the characteristics of included studies, with respect to the item focusing on the presence of “objective, standard criteria for measurement of the condition”, we considered that the authors satisfied the condition when they directly referred and specifically defined the populations’ migrant status.

With respect to confounding factors and strategies to deal with them, we considered whether if authors provided adequate information on baseline characteristics and whether they took the confounding factors (age, sex, country of origin, etc.) into account by considering them for adjustment or stratification in statistical analyses.

When considering longitudinal studies, some of the items provided in the tool for cohort studies did not apply to the characteristics of included studies, so they were labelled as “not applicable”.
